# High β-Lactam and Quinolone Resistance of *Enterobacteriaceae* from the Respiratory Tract of Sheep and Goat with Respiratory Disease

**DOI:** 10.3390/ani11082258

**Published:** 2021-07-30

**Authors:** Hazim O. Khalifa, Atef Oreiby, Amer Ali Abd El-Hafeez, Amira Abd El Latif, Takashi Okanda, Yasuyuki Kato, Tetsuya Matsumoto

**Affiliations:** 1 Department of Infectious Diseases, Graduate School of Medicine, International University of Health and Welfare, Narita 286-8686, Japan; katoy@iuhw.ac.jp (Y.K.); tetsuya.m@iuhw.ac.jp (T.M.); 2 Department of Pharmacology, Faculty of Veterinary Medicine, Kafrelsheikh University, Kafr El-Sheikh 33516, Egypt; amirashehata12@yahoo.com; 3 Division of Clinical Research, Medical Mycology Research Center, Chiba University, Chiba 260-8673, Japan; 4 Department of Animal Medicine (Infectious Diseases), Faculty of Veterinary Medicine, Kafrelsheikh University, Kafr El-Sheikh 33516, Egypt; atef.ibrahim@vet.kfs.edu.eg; 5 Pharmacology and Experimental Oncology Unit, Cancer Biology Department, National Cancer Institute, Cairo University, Cairo 12613, Egypt; amer.ali@nci.cu.edu.eg; 6 Department of Cellular and Molecular Medicine, School of Medicine, University of California, San Diego, CA 92093-0651, USA; 7 Department of Microbiology, St. Marianna University School of Medicine, Sugao, Kawasaki 216-8511, Japan; takashi.okanda@marianna-u.ac.jp

**Keywords:** multidrug-resistance, AmpC beta-lactamase, extended-spectrum beta-lactamase, quinolone resistance, Egypt, sheep and goats

## Abstract

**Simple Summary:**

β-lactams and quinolones are major groups of antibiotics that are commonly used for the treatment of severe infection both in animals and humans. Little is known about their resistance mechanisms in animals. Our results revealed high resistance rates against both groups in Gram-negative bacteria recovered from small ruminants suffering from respiratory disease. Phenotypically, 9.2% of the isolates were multidrug-resistant, and 11.8% and 6.6% of the isolates were positive for AmpC and ESBL production, respectively. Genetic characterization identified different β-lactamase-encoding genes such as *bla*_TEM_, *bla*_SHV_, and *bla*_CTX-M_ which are responsible for β-lactam resistance. Furthermore, the plasmid-mediated quinolone resistance gene, *qnrS*, was identified to be associated with quinolone resistance. Our results regenerate interest in the wise use of antimicrobials in animal fields as well as to apply a One Health approach to prevent and/or mitigate their dissemination to the human environment.

**Abstract:**

During the last decade’s increase of antimicrobial resistance (AMR) in animals, animal-human transmission has become a major threat. Therefore, the present study aimed to evaluate the genetic basis of AMR in Gram-negative bacteria recovered from sheep and goats with respiratory disease. Nasal and ocular swabs were collected from 69 diseased animals, and 76 Gram-negative bacterial isolates were identified from 59 animals. All isolates were checked phenotypically for resistance and genotypically for different resistance mechanisms, including β-lactam, quinolone, and aminoglycoside resistance. Our results demonstrated that 9.2% (95% CI 4.5–17.8%) of the isolates were multidrug-resistant, with high resistance rates to β-lactams and quinolones, and 11.8% (95% CI 6.4–21%) and 6.6% (95% CI 2.8–14.5%) of the isolates were phenotypically positive for AmpC and ESBL, respectively. Genotypically, *bla*_TEM_ was the most identified β-lactamase encoding gene in 29% (95% CI 20–40%) of the isolates, followed by *bla*_SHV_ (14.5%, 95% CI 8.3–24.1%) and *bla*_CTX-M_ (4%, 95% CI 1.4–11%). Furthermore, 7.9% (95% CI 3.7–16.2%) of the isolates harbored plasmid-mediated quinolone resistance gene *qnrS*. Our study revealed for the first time to our knowledge high β-lactam and quinolone resistance associated with the bacteria recovered from sheep and one goat with respiratory disease. Furthermore, different antimicrobial resistant determinants were identified for the first time from animals in Africa, such as *bla*_LEN-13/55_, *bla*_TEM-176_ and *bla*_TEM-198/214_. This study highlights the potential role of sheep and goats in disseminating AMR determinants and/or resistant bacteria to humans. The study regenerates interest for the development of a One Health approach to combat this formidable problem.

## 1. Introduction

In recent years, the worldwide accretion of antimicrobial resistance (AMR) has gained great attention. Increased AMR is associated with the heavy and imprudent use of antimicrobials in humans and animals, for therapeutic and nontherapeutic implications [1]. The dilemma of AMR has extended its health burden to also include the economic costs. According to a recent appraisal, AMR is responsible for GBP 3–11 billion to USD 100 trillion in economic losses [2]. Recently, there have been growing reports that elucidate and support the potential link between the emergence of AMR in humans and AMR in livestock populations [2,3]. Gram-negative bacteria, especially *Enterobacteriacea,* are of grave concern as their emergence and transmission, particularly extended-spectrum beta-lactamase (ESBL) and AmpC-producers from animals, have become a critical worldwide community concern [4]. The uncontrolled use of antibiotics in humans and animals, coupled with the use of human antibiotics in veterinary medicine has resulted in increasing the rates of AMR, especially in developing countries including Egypt [5,6,7,8,9,10,11,12].

β-lactam and quinolones are classes of antibiotics widely used and classified as “critically important” for human use by the WHO [13]. However, their extensive use in humans and animals results in the emergence and spread of their resistance [6,7]. β-lactam resistance is mediated by the ability of bacteria, especially Gram-negative bacteria that produce β-lactamases [6,7]. Among β-lactamases, extended-spectrum and AmpC beta-lactamases (ESBL/AmpC) have drawn much attention [6,7]. These enzymes hydrolyze a wide range of β-lactams and induce resistance to different antimicrobial drugs, which is consequently associated with treatment failure in humans and animals. Furthermore, recent poor therapeutic outcomes with substantially high mortality rates were associated with ESBL in humans [7].

Quinolone resistance is attributed to mutations in the drug’s target enzymes DNA gyrase and DNA topoisomerase IV and mutations in bacterial membrane efflux pump regulatory genes [14]. Furthermore, the bacterial acquisition of resistance genes, such as plasmid-mediated resistance gene *qnr*, are also responsible for low resistance levels [6,7,14]. However, these genes have gained a special interest owing to their rapid bacterial dissemination by plasmid mobility and their presence might potentiate the incidence of high resistance levels [6,7,14].

*Enterobacteriaceae* acquire ESBL genes as well as other antimicrobial resistance determinates by mutation or horizontal gene transfer, which allows the rapid transmission and dissemination of antimicrobial resistance [4]. The emergence of ESBL-producing *Enterobacteriaceae* has been well documented in animals and/or animal products in Europe and Asia [1,2,3,4]. However, little is known about their prevalence and their genetic basis of AMR in animals in Egypt. Therefore, the present study was designed to evaluate the prevalence and genetic basis of AMR in *Enterobacteriaceae* recovered from the respiratory tracts of sheep and goats infected with respiratory infections in Egypt. To the best of our knowledge, this is the first African study elucidating AMR genetic mechanisms associated with *Enterobacteriaceae* recovered from sheep and goats with respiratory infections.

## 2. Materials and Methods

### 2.1. Animals and Sampling

In this study, between November and December 2017 from seven different private and governmental farms in Kafrelsheikh city, Egypt, a total of 69 deep nasal swabs and three ocular swabs were collected from 69 diseased animals (65 sheep and 4 goats) after careful clinical examination for respiratory signs. The diseased animals were suffering from different manifestations such as cough, pneumonia, and fever. Strict aseptic conditions were applied for collection and transmission of the swabs to the lab at 4 °C for further microbiological and molecular analyses.

### 2.2. Bacterial Isolation and Identification

For *Enterobacteriaceae* isolation and identification, the swabs were enriched under aseptic conditions in 5 mL tryptic soya broth at 37 °C for 16–18 h, followed by streaking on MacConkey agar plates. After 24 h incubation at 37 °C, suspected colonies were isolated and preserved at 20% glycerol stocks for further investigation. A total of 76 non-duplicate Gram-negative bacteria were recovered from the nasal cavity of 59 diseased animals (58 sheep and 1 goat), with 43 animals harboring a single bacterial infection and 16 animals harboring multiple bacterial infections. Matrix assisted laser desorption ionization-time of flight mass spectrometry (MALDI-TOF-MS) was used for isolate identification.

### 2.3. Antimicrobial Sensitivity Testing

Antimicrobial susceptibility of the isolates was tested by the Kirby–Bauer disc diffusion method according to the Clinical and Laboratory Standards Institute (CLSI) [15]. In this study, different classes of antibiotics were tested, including β-lactams (amoxicillin–clavulanic acid [AMC], 20–10 µg; ampicillin [AMP], 10 µg; cefoperazone [CFP], 75 µg; ceftriaxone [CRO], 30 µg; cefoxitin [FOX], 30 µg; imipenem [IPM], 10 µg; and meropenem [MEM]; 10 µg); quinolones (nalidixic acid [NAL], 30 µg and ciprofloxacin [CIP], 5 µg); aminoglycosides (amikacin [AMK], 30 µg and gentamicin [GEN],10 µg); chloramphenicol (CHL), 30 µg; and tetracycline (TET), 30 µg. All discs were purchased from Becton Dickinson and Company Sparks (Sparks, MD 21152, USA).

### 2.4. Detection of Carbapenemase Production

A modified carbapenem inactivation method was applied for the detection of carbapenemase production in isolates as previously described [16]. In brief, under aseptic conditions, 1 μL inoculation loop of the tested isolate previously cultured overnight on a nutrient agar (Eiken Chemical Co., LTD., Tochigi, Japan) at 37° C was added to a tube containing 2 mL of tryptic soy broth (Becton, Dickinson and Company, Sparks, MD 21152 USA). The bacterial suspension was vortexed for 15 sec, then a 10 μg MEM disc was added, followed by 4 h incubation at 37 °C. *E. coli* ATCC 25922 at a concentration of 0.5 McFarland standard was used as an indicator organism and thoroughly spread on a Muller Hinton agar (MHA, Becton, Dickinson and Company, Sparks, MD 21152 USA) plate using a sterile cotton swab before the end of the incubation time. After 4 h incubation with the MEM disk and bacterial suspension, the disk was aseptically removed and placed on the inoculated MHA plate, followed by incubation for 18–24 h at 37 °C. Inhibition zone diameters around the MEM discs were measured after the incubation time. *E. coli* ATCC 25922 was used as a negative control, and carbapenemase-producing isolates from our previous reports served as positive controls [5,8,9].

### 2.5. Detection of ESBL and AmpC production

In this study, D68C AmpC and ESBL detection set (Mast Diagnostics, Mast Group Ltd., Merseyside, UK) were used to detect ESBL and AmpC production in bacterial isolates as per the manufacturer’s instructions and as previously reported [6]. Briefly, the tested isolates at concentrations of 0.5 McFarland standard were inoculated on MHA plates after overnight culture at 37 °C. The four discs included in the set were aseptically placed on the inoculated MHA plates and incubated for 24 h at 37 °C. After the incubation time, inhibition zone diameters around the discs were measured, and the results were evaluated by comparing the inhibition zone diameters of the four discs. Of note, the four discs included in the D68C AmpC and ESBL detection set were: disc A that contains cephalosporin (10 μg cefpodoxime), disc B that contains cephalosporin and ESBL inhibitor (clavulanate), disc C that contains cephalosporin and AmpC inhibitor (cloxacillin), and disc D that contains cephalosporin and both AmpC and ESBL inhibitors. In this test, *E. coli* ATCC 25922 was used as a non-ESBL and non-AmpC producer, while ESBL and AmpC producers from our previous reports were used as positive controls [6].

### 2.6. DNA Preparation for PCR Experiments

The DNA was prepared using DNA extraction kits (Cica Geneus DNA extraction reagent, KANTO CHEMICAL CO., INC., Tokyo, Japan) according to manufacturer’s instructions. Briefly, 10 μL of overnight cultured bacterial suspension at a concentration of 1– 3 McFarland standard was mixed with 100 µL of kit reagents, followed by incubation at 72 °C for 6 min and at 94 °C for 4 min. The bacterial suspension was thoroughly mixed and centrifuged at 15,000× *g* rpm for 1 min, and the supernatant was used as a template for PCR experiments.

### 2.7. Molecular Screening for ESBL and AmpC-Encoding Genes

Multiplex PCR followed by single PCR for positive isolates and sequencing were employed to detect ESBL and/or AmpC-encoding genes and other β-lactamases as previously described [17,18,19] (Table 1). The isolates were tested for the presence of *bla*_CTX-M_, *bla*_SHV_, and *bla*_TEM_ to confirm the β-lactams resistance and ESBL production. Furthermore, all the isolates were assessed for the presence of *bla*_ACC_, *bla*_LAT_, *bla*_CMY_, *bla*_BIL_, *bla*_MOX_, *bla*_DHA_, *bla*_MIR_, *bla*_ACT_, and *bla*_FOX_ to confirm AmpC production.

### 2.8. Molecular Screening for Quinolone and Aminoglycoside Resistance

Quinolone resistance was evaluated by testing all the isolates for the presence of plasmid-mediated quinolone resistance genes (*qnrA*, *qnrB*, and *qnrS*) and quinolone efflux pump determinant, *qepA*, as previously described [20,21] (Table 1). Aminoglycoside resistance was assessed by determining the presence of 16S rRNA methylases (*rmtA*, *rmtB*, *rmtC*, *rmtD*, *armA*, and *npmA*) as previously described [22] (Table 1).

### 2.9. Sequencing and Sequence Data Analysis

PCR reaction products were analyzed by 1.0–2.0% agarose gel electrophoresis; then, gels were stained with ethidium bromide and visualized under ultraviolet light. The Cica Geneus PCR & Gel Prep Kit (KANTO CHEMICAL CO., INC., Tokyo, Japan) was used for PCR product purification according to the manufacturer’s instructions. The sequencing of PCR products was performed by ABI 3130xl Genetic Analyzer and the BigDye Terminator Cycle Sequencing Kit, v3 as previously described [23,24]. After sequencing, BLAST analysis (http://blast.ncbi.nlm.nih.gov/Blast.cgi (accessed on 10–26 January 2021)) was performed to assess similarities among the sequenced data.

### 2.10. Statistical Analysis

The correlation between the bacterial isolates, phenotypic, and genotypic data were analyzed by Fisher’s exact test, followed by a post hoc pairwise comparisons test using R-statistical software. The confidence interval of the proportion of all the findings among examined animals was calculated as follows: p ± z√((p(1 − p))/n). Where p is the proportion of animals showing the special findings, n is the number of examined animals and z is the level of confidence and it equals 1.96 for 95% confidence.

## 3. Results

### 3.1. Isolates Identification

**Figure 1:**
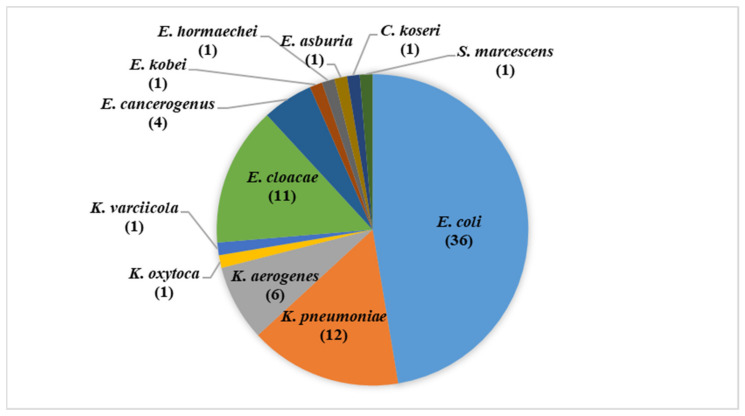
Bacterial isolates identified and tested in this study.

Out of the 1050 animals, there were 69 disease cases, with a sampling fraction of 6.6% (95% CI 5.2–8.3%). Seventy-six Gram-negative bacteria were recovered from 59 disease cases with a percentage of 85.5% (95% CI 75.3–92.8%). The identified isolates were: 36 *Escherichia coli* isolates (47.4%, 95% CI 36.54–58.45%), 20 *Klebsiella* spp. isolates (26.3%, 95% CI 16.9–37.7%), 18 *Enterobacter* spp. isolates (23.7%, 95% CI 14.7–24.8%), and a single *Citrobacter koseri*, and *Serratia marcescens* isolates (1.3%, 95% CI 0.2–7.1% each) (Figure 1). *Klebsiella* spp. include 12 *K. pneumoniae* isolates, six *K. aerogenes* isolates, a single isolate of *K. oxytoca*, and a single isolate *K. varciicola*. *Enterobacter* spp. include 11 *E. cloacae* isolates, four *E. cancerogenus* isolates, a single isolate of either *E. kobei*, *E. hormaechei* or *E. asburia*.

### 3.2. Phenotypic Characterization of Isolates

**Figure 2:**
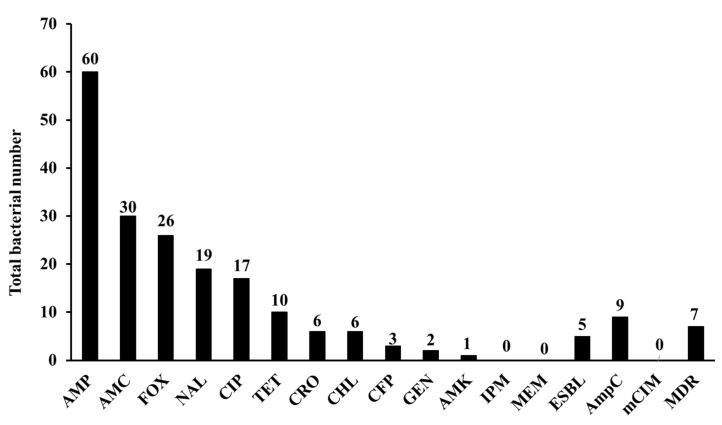
Level of antimicrobial resistance to different antibiotics and phenotypic characteristics of the isolates. Antibiotics: ampicillin (AMP), amoxicillin–clavulanic acid (AMC), cefoxitin (FOX), nalidixic acid (NAL), ciprofloxacin (CIP), tetracycline (TET), ceftriaxone (CRO), chloramphenicol (CHL), cefoperazone (CFP), gentamicin (GEN), amikacin (AMK), imipenem (IPM), and meropenem (MEM).

In this study, 9.2% (95% CI 4.5–17.8%) of the isolates showed multidrug-resistant profiles attributed to the resistance to three or more antimicrobial classes (Figure 2, Supplementary Table S1). The isolates showed high resistance rates to AMP, AMC, FOX, NAL, and CIP, 78.9% (95% CI 68.5–86.6%), 39.5% (95% CI 29.2–50.7%), 34.2% (95% CI 24.5–45.4%), 25% (95% CI 16.6–35.8%), and 22.4% (95% CI 14.5–33%), respectively. On the other hand, the isolates showed low resistance rates to TET (13.2%, 95% CI 7.3–22.6%), CRO (7.9%, 95% CI 3.7–16.2%), CHL (7.9%, 95% CI 3.7–16.2%), CFP (3.9%, 95% CI 1.4–11%), GEN (2.6%, 95% CI 0.7–9.1%), and AMK (1.3%, 95% CI 0.2–7.1%). All the isolates were sensitive to IPM and MEM (Figure 2, Supplementary Table S1).

Phenotypic AmpC production was identified in 11.8% (95% CI 6.4–21%) of the isolates, and 6.6% (95% CI 2.8–14.5%) of the isolates produced ESBL. None of the isolates produced carbapenemases as determined by the mCIM assay (Figure 2, Supplementary Table S1).

### 3.3. Prevalence of β-Lactamase Encoding Genes

**Figure 3:**
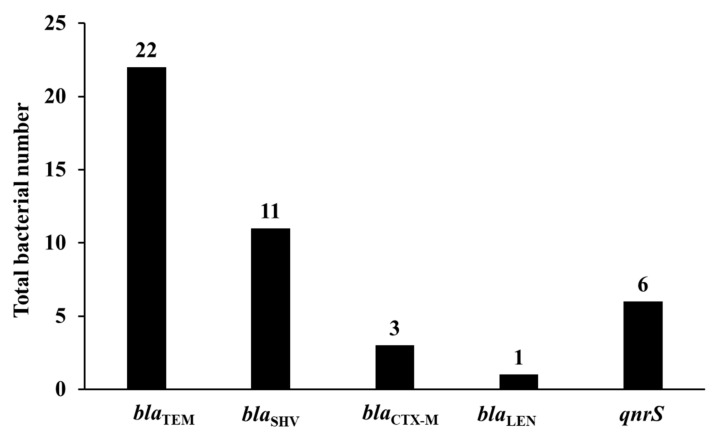
Detected antimicrobial resistance genes. Regarding β-lactamase encoding genes, *bla*_TEM_ was identified in 22 isolates (29% 95% CI 20–40%) of the isolates, followed by *bla*_SHV_ identified in 11 isolates (14.5%, 95% CI 8.3–24.1%), *bla*_CTX-M_ identified in three isolates (4%, 95% CI 1.4–11%), and finally, a single isolate harbored *bla*_LEN-13/55_ (1.3%, 95% CI 0.2–7.1%). *qnrS* was the only identified PMQR in six isolates (7.9%, 95% CI 3.7–16.2%).

Our results confirmed that *bla*_TEM_ was the most prevalent β-lactamase encoding gene identified in 29% (95% CI 20–40%) of the isolates with 17 isolates harboring *bla*_TEM-1_, three isolates harboring *bla*_TEM-176_, and two isolates harboring *bla*_TEM-198/214_. *bla*_SHV_ was the second most identified β-lactamase encoding genes in 14.5% (95% CI 8.3–24.1%) of the isolates with 10 isolates harboring *bla*_SHV-1_ and a single isolate harboring *bla*_SHV-1/11_. *bla*_CTX-M_ was identified only in three isolates (4%, 95% CI 1.4–11%), with two isolates harboring *bla*_CTX-M-15-like_ and a single isolate harboring *bla*_CTX-M-14_ (Figure 3, Supplementary Table S1). Interestingly, a single isolate was positive for *bla*_TEM_ by PCR using TEM primers, but after sequencing it was confirmed that it harbored *bla*_LEN-13/55_. Despite of using different sets of primers, conditions, PCR reagents, and repeating the experiments all the isolates were genotypically negative for plasmid AmpC-encoding genes.

### 3.4. Prevalence of Plasmid-Mediated Quinolone Resistance Genes and Other Resistance Genes

In our study, only *qnrS* was the identified PMQR in 7.9% (95% CI 3.7–16.2%) of the isolates (Figure 3, Supplementary Table S1). Multiplex and/or single PCR confirmed that none of the isolates harbored *qepA* and 16S rRNA methylases genes.

### 3.5. Relation between the Recovered Isolates and Resistant Determinants

There was no relation between the recovered bacterial isolate and incidence of multidrug resistance infection (*p* = 0.4), incidence of infection with ESBL-producers (*p* = 0.6), identification of *bla*_CTX-M_ (*p* = 0.8), or *qnrS* identification (*p* = 0.6). On the other hand, the recovered bacterial isolates were statistically related to the incidence of infection with AmpC-producing isolates (*p* = 0.01). Furthermore, there was a statistical relationship between the recovered bacterial isolates and identification of *bla*_SHV_ gene (*p* = 0.000008) with most *bla*_SHV_ genes identified in *K. pneumoniae*, and the identification of *bla*_TEM_ gene (*p* = 0.0002), with most *bla*_TEM_ gene identified in *E. coli* isolates.

## 4. Discussion

The extensive use of antimicrobials in the veterinary field for the treatment and prevention of infectious diseases and to increase animal production has led to the dissemination of antimicrobial resistance. Furthermore, the close contact between animals and humans facilitates cross-transmission, especially members of the *Enterobacterales* which are closely related normal intestinal flora that rarely cause disease in normal hosts. However, they can cause serious disease in immunocompromised patients due to other diseases [4], and have the ability to acquire and disseminate several unique resistance mechanisms [25,26]. The emergence and spread of ESBL- and Amp-producing *Enterobacteriaceae* from animals has become an important community concern worldwide. Unfortunately, data are lacking about the molecular resistance mechanisms associated with *Enterobacteriaceae* from animals, especially sheep and goats in Egypt. Therefore, this study heightened the genetic mechanisms governing these isolates in sheep and goats.

In this study, 9.2% of the isolates were multidrug-resistant, which is comparable or lower than previous findings of primary respiratory pathogens belonging to the *Pasteurellaceae* family in Canada that ranged from 11.8% to 38.1% [27]. On the other hand, our results showed very high resistance levels for different β-lactams, including AMP (78.9%), AMC (39.5%), and FOX (34.2%). Our results were higher than those from previous reports of sheep and goat respiratory tract pathogens in Canada [27] and the USA [28]. Interestingly, our results for the *Enterobacteriaceae* showed a high resistance rate also for quinolones, with 25% and 22.4% of isolates resistant to NAL and CIP, respectively. These were higher frequencies than those previously reported from North American countries [27,28] and some Asian and African countries [29,30]. Most of the isolates were susceptible to tetracycline, aminoglycosides, and chloramphenicol. Similar tetracycline antimicrobial susceptibility patterns have been reported in other studies from *Pasteurellaceae* in North America [27,28]. However, high tetracycline resistance has been identified in *Mycoplasma mycoides* subspecies *capri* in Pakistan and respiratory tract bacterial flora in Nigeria [29,30]. The high prevalence of β-lactam and quinolone resistance in Egypt is an issue of great clinical consideration as these drugs are commonly used for the treatment of severe infections in both animals and humans [6,31]. Although a high prevalence of carbapenem resistance and carbapenemase production has been observed in clinical settings in Egypt [8,9], all isolates were sensitive to carbapenem antibiotics and phenotypically negative for carbapenemase production (Figure 2, Supplementary Table S1). This may be attributed to the restrictions of carbapenem use in the veterinary field in Egypt. Carbapenems are clinically important lifesaving β-lactams, and this study rejuvenated the concern for their constraint in veterinary and agriculture fields.

To understand the genetic basis of β-lactam resistance, the isolates were assessed phenotypically for ESBL and AmpC production and genotypically different classes of β-lactamases. Although ESBL and AmpC production are considered as major mechanisms of β-lactams resistance [6,7,31], only 11.8% and 6.6% of the isolates were phenotypically positive for AmpC and ESBL production, respectively. On the other hand, with the exception of the identified *bla*_CTX-M_, genotypic examination identified different narrow-spectrum β-lactamases such as *bla*_SHV_ and *bla*_TEM_. The high prevalence of β-lactamases in our study explaining the high levels of resistance AMP, AMC, and FOX. Our study showed that *bla*_TEM_ and *bla*_SHV_ were the most abundant β-lactamases which was in agreement with the previous reports from other developing countries such as ESBL-producing *E. coli* in Turkey [32]. More interestingly, in this study, several antibiotic resistant determinants were identified for the first time from animals in Egypt or even in Africa, such as *bla*_LEN-13/55_, *bla*_TEM-176_ and *bla*_TEM_-_198/214_. Furthermore, this is the first report of *bla*_CTX-M_ identification in small ruminants in Egypt. Notably, CTX-M enzymes have recently emerged in different isolates from food, food-producing animals, and companion animals [5,31,32,33,34], triggering their potential as a public health hazard. For instance, different variants of *bla*_CTX-M_, including *bla*_CTX-M-1_, *bla*_CTX-M-2_, *bla*_CTX-M-3_, *bla*_CTX-M-9_*, bla*_CTX-M-14_, *bla*_CTX-M-15_, and *bla*_CTX-M-28_, have been identified from diseased and healthy animals and/or food products in Spain, Denmark, France, Netherlands, UK, China, and Japan [31,35]. Furthermore, *bla*_CTX-M_ was the most identified ESBL gene in Egypt from clinical settings and food [7,34], indicating potential transmission among animals, humans, and food. Although 11.8% (95% CI 6.4–21%) of the isolates were phenotypically positive for AmpC production, no plasmid-mediated AmpC beta-lactamase genes were detected, despite repeating the PCR experiment with using different sets of primers and different conditions (data not shown). Other mechanisms, especially the chromosomal AmpC which are intrinsic in certain bacterial isolates such as *Enterobacter* and *Citrobacter* spp. might be responsible for the phenotypic resistance to AmpC. Confirming our findings, plasmid AmpC was recently identified in only 1.1% of sheep meat samples in Egypt [36].

In this study, 7.9% (95% CI 3.7–16.2%) of the isolates harbored the *qnrS* gene, confirming our previous results that *qnrS* is the major circulating PMQR gene in Egypt in clinical, veterinary, and food production settings [7,34]. Much higher than our results, *qnrS* was the only detected PMQR gene identified in 44% of ESBL-producing *E. coli* from companion and domestic farm animals in Tanzania [33]. Furthermore, *qnrS* was only detected in sheep in Italy and was the most prevalent among *Salmonella* and *E. coli* isolates from animals, humans, food, and the environment from 13 European countries [37]. *qnrS* was also the only PMQR gene detected in 80% of ESBL-producing *Enterobacteriaceae* recovered from sheep in Abidjan, Ivory Coast [38]. On the other hand, *qnrB*, *qnrA*, and *qnrS* were recently identified in *E. coli* from healthy and diarrheic sheep and goats in Saudi Arabia by 62.1, 56, and 52.4%, respectively [39]. In a recent study from Tunisia, although *qnrS1* and *qnrB1* were identified among the *E. coli* recovered from poultry and turkeys, no PMQR genes were identified from sheep [40]. The prevalence of *qnrS* identified in this study is of considerable clinical impact, as PMQR genes not only potentiate quinolone resistance, they also disseminate quinolone resistance through their plasmid mobility [6,13]. Our results confirmed the limited role of *qepA* and 16S rRNA methylases to mediate quinolone and aminoglycoside resistance, respectively, which is also in agreement with our previous report from food and pets in Egypt [6,34].

As a limitation of this study that only four goats were checked, and a single isolate was recovered from one goat and wild small ruminants were not included. Furthermore, this study was performed in a single city in Egypt. Therefore, it is of critical importance to perform other large-scale nationwide studies on a wide range of small ruminants to evaluate the molecular resistance mechanisms all over Egypt. Even so, this study is considered to be the first African study to evaluate the AMR associated with Gram-negative bacteria recovered from the respiratory tract of sheep and goats with respiratory infections.

## 5. Conclusions

This study identified *Enterobacteriaceae* recovered from sheep and one goat suffering from respiratory infections in Egypt, highlighted the prevalence of β-lactam and quinolone resistance. The high prevalence of antimicrobial resistance in sheep and goats is an issue of grave concern given the opportunity of human transmission and development of severe, untreatable infections. Therefore, strict regulations such as prudent antibiotic use, large scale surveillance, and prompt infection control measures are necessary to combat this problem.

## Figures and Tables

**Table 1:** Oligonucleotides used in this study.

Primer Name	Sequence (5’-3’)	Amplicon Size (bp)	Target	Reference
β-lactamases				
CTXM7 CTXM8	GCGTGATACCACTTCACC TC TGAAGTAAGTGACCAGAA TC	260	*bla*_CTX-M-1group_	[17]
CTXM17 CTXM18	TGATACCACCACGCCGCT C TATTGCATCAGAAACCGTGGG	341	*bla*_CTX-M-2group_	[17]
CTXM19 CTXM20	CAATCTGACGTTGGGCAATG ATAACCGTCGGTGACAATT	207	*bla*_CTX-M-8/25/26group_	[17]
CTXM11 CTXM12	ATCAAGCCTGCCGATCTGGTTA GTAAGCTGACGCAACGTCTGC	293	*bla*_CTX-M-9group_	[17]
SHV_F SHV_R	AGCCGCTTGAGCAAATTAAAC ATCCCGCAGATAAATCACCAC	713	*bla*_SHV-1/variant_	[18]
TEM-F TEM-R	CATTTCCGTGTCGCCCTTATTC CGTTCATCCATAGTTGCCTGAC	800	*bla*_TEM-1/-2/variant_	[18]
MOXMF MOXMR	GCT GCTCAAGGAGCACAG GAT CAC ATT GAC ATA GGT GTG GTG C	520	*bla*_MOX-1,_*bla*_MOX-2,_ and *bla*_CMY-1,_ _CMY-8 to CMY-11_	[19]
CITMF CITMR	TGGCCAGAACTGACAGGCAAA TTTCTCCTGAACGTGGCTGGC	462	*bla*_LAT-1 to LAT-4,_*bla*_CMY-2_ _to CMY-7,_ and *bla*_BIL-1_	[19]
DHAMF DHAMR	AACTTTCACAGGTGTGCTGGGT CCGTACGCATACTGGCTTTGC	405	*bla*_DHA_	[19]
ACCMF ACCMR	AACAGCCTCAGCAGCCGGTTA TTCGCCGCAATCATCCCTAGC	346	*bla*_ACC_	[19]
EBCMF EBCMR	TCGGTAAAGCCGATGTTGCGG CTTCCACTGCGGCTGCCAGTT	302	*bla*_MIR-1_ and *bla*_ACT-1_	[19]
FOXMF FOXMR	AACATGGGGTATCAGGGAGATG CAAAGCGCGTAACCGGATTGG	190	*bla*_FOX-1 to FOX-5b_	[19]
Plasmid-mediated quinolone resistance				
qnrA-F	ATTTCTCACGCCAGGATTTG	468	*qnrA*	[20]
qnrA-R	TGCCAGGCACAGATCTTGAC			
qnrB-F	CGACCTKAGCGGCACTGAAT	513	*qnrB*	[20]
qnrB-R	GAGCAACGAYGCCTGGTAGYTG			
qnrS-F	ACTGCAAGTTCATTGAACAG	431	*qnrS*	[20]
qnrS-R	GATCTAAACCGTCGAGTTCG			
Quinolone efflux pump determinant				
qepA-F qepA-R	AACTGCTTGAGCCCGTAGAT GTCTACGCCATGGACCTCAC	596	*qepA*	[21]
16S rRNA methylases				
armA-F armA-R	GGTGCGAAAACAGTCGTAGT TCCTCAAATATCCTCTATGT	1153	*armA*	[22]
npmA-F npmA-R	CGGGATCCAAGCACTTTCATACTGACG CGGAATTCCAATTTTGTTCTTATTAGC	981	*npmA*	[22]
rmtA-F rmtA-R	CTAGCGTCCATCCTTTCCTC TTTGCTTCCATGCCCTTGCC	635	*rmtA*	[22]
rmtB-F rmtB-R	GGAATTCCATATGAACATCAACGATGCC CCGCTCGAGTCCATTCTTTTTTATCAAGT	756	*rmtB*	[22]
rmtC-F rmtC-R	CGAAGAAGTAACAGCCAAAG GCTAGAGTCAAGCCAGAAAA	1000	*rmtC*	[22]
rmtD-F rmtD-R	TCATTTTCGTTTCAGCAC AAACATGAGCGAACTGAAGG	744	*rmtD*	[22]

## Data Availability

All the data are presented in this study and in the Supplementary File.

## Supplementary Materials

The following are available online at https://www.mdpi.com/article/10.3390/ani11082258/s1, Table S1: Full phenotypic and genotypic characterization of the isolates tested in this study.
