# Supplementary material for: High β-Lactam and Quinolone Resistance of Enterobacteriaceae from the Respiratory Tract of Sheep and Goat with Respiratory Disease
Source: Animals (Basel). 2021 Jul 30;11(8):2258. doi: 10.3390/ani11082258 (PMC8388476; doi:10.3390/ani11082258)
Supplement: Supplementary file 1 [file animals-11-02258-s001.zip › animals-1265321-supplementary.pdf]

Supplementary Table S1. Full phenotypic and genotypic characterization of the isolates tested in this study

No	Strain	Species	Resistance Phenotype	MDR phenotype	ESBL	AmpC	*bla genes*	*qnr genes*
1.	3R-R1	*E. coli*	AMP					
2.	3R-R2	*K. pneumoniae*	AMP, TET				*bla*$_{SHV-1}$	
3.	4R	*E. coli*	AMP				*bla*$_{TEM-1}$	
4.	6R	*K. oxytoca*	AMC, AMP, CRO					
5.	7R	*E. cloacae*	AMC, AMP, FOX, TET					
6.	8R1	*E. coli*	AMC, AMP, CHL, CIP, NAL	+			*bla*$_{TEM-198/214}$	
7.	8R2	*E. hormaechei*	AMP, FOX				*bla*$_{SHV-1}$	
8.	9R	*K. pneumoniae*	AMP, FOX				*bla*$_{SHV-1}$	
9.	10R	*E. cloacae*	AMC, AMP, FOX					
10.	11R	*E. cloacae*	AMP					
11.	12R	*E. cloacae*	AMC, AMP, FOX			+		
12.	13R-R	*E. cloacae*	AMC, AMP, FOX			+		
13.	14R-R	*K. variicola*	AMP				*bla*$_{SHV-1}$	
14.	14R-W	*E. asburiae*	AMC, AMP, FOX			+		
15.	15R	*E. coli*						
16.	16R	*E. cloacae*	AMC, AMP, FOX					
17.	17R	*E. cloacae*	AMC, AMP, FOX			+		
18.	18R	*E. cloacae*	AMC, AMP, FOX					
19.	19R	*E. coli*	AMP, CFP, CRO		+		*bla*$_{CTX-M-15-like}$	
20.	20R-R	*E. coli*	AMP, CRO, TET		+		*bla*$_{TEM-1B}$	
21.	23R	*E. coli*						
22.	24R	*S. marcescens*	AMC, AMP, FOX			+		
23.	25RT	*E. coli*						
24.	25RL	*K. aerogenes*	AMC, AMP, FOX					

25.	26R	*E. coli*	AMP, CRO, FOX					
26.	27R	*K. pneumoniae*	AMP					
27.	28R	*E. coli*						
28.	29R	*E. coli*						
29.	30R	*E. coli*	AMK					
30.	31R-R	*E. coli*						
31.	31R-W	*E. kobei*	AMC, AMP, FOX					
32.	32R	*E. cloacae*	AMC, AMP, FOX					
33.	33R	*K. aerogenes*	AMC, AMP, FOX					
34.	34R	*K. aerogenes*	AMC, AMP, FOX					
35.	35R	*E. cancerogenus*	AMC, AMP, FOX					
36.	36R	*K. aerogenes*	AMC, AMP, FOX					
37.	37R1	*K. pneumoniae*	AMC, AMP				*bla*$_{\text{SHV-1}}$	
38.	37R2	*E. coli*						
39.	38R1	*E. coli*						
40.	38R2	*K. pneumoniae*	AMC, AMP, GEN, TET	+			*bla*$_{\text{SHV-1}}$, *bla*$_{\text{TEM-1}}$	*qnrS1*
41.	39R1	*E. coli*						
42.	39R2	*K. pneumoniae*					*bla*$_{\text{LEN-13/55}}$	
43.	40R-R	*K. pneumoniae*	AMP				*bla*$_{\text{SHV-1/11}}$	
44.	40R-W	*C. koseri*	AMC, AMP, FOX			+		
45.	43R-R	*E. coli*						
46.	43R-W	*E. cloacae*	AMC, AMP, FOX			+		
47.	44R	*K. pneumoniae*	AMP				*bla*$_{\text{SHV-1}}$	
48.	45R	*K. pneumoniae*	AMP				*bla*$_{\text{SHV-1}}$	
49.	46R1	*K. pneumoniae*	AMP				*bla*$_{\text{SHV-1}}$	
50.	46R2	*K. aerogenes*	AMC, FOX					
51.	47R1	*E. coli*	AMC, AMP, CFP, CHL, CIP, CRO, GEN, NAL, TET	+	+		*bla*$_{\text{CTX-M-14}}$, *bla*$_{\text{TEM-1B}}$	

52.	47R2	*K. aerogenes*	AMC, FOX					
53.	48R	*E. coli*	AMP, CIP, NAL				$bla_{\text{TEM-1}}$	
54.	51R	*E. coli*	AMP, CIP, NAL				$bla_{\text{TEM-1}}$	
55.	52R	*E. coli*	AMP, CIP, NAL				$bla_{\text{TEM-1}}$	
56.	53R	*E. coli*	AMP, CIP, CHL, NAL, TET	+			$bla_{\text{TEM-176}}$	*qnrS1*
57.	54R	*E. coli*	AMP, CIP, NAL				$bla_{\text{TEM-1}}$	
58.	55R1	*K. pneumoniae*					$bla_{\text{SHV-1}}$	
59.	55R2	*K. pneumoniae*	AMP, CIP, NAL					
60.	56R-R	*E. coli*	AMP, CIP, NAL				$bla_{\text{TEM-1}}$	
61.	56R-W	*E. cancerogenus*	AMC, AMP			+		
62.	57R	*E. coli*	AMP, CHL, NAL, TET	+			$bla_{\text{TEM-1}}$	*qnrS1*
63.	58R	*E. coli*	AMP, CIP, NAL				$bla_{\text{TEM-1}}$	
64.	59R	*E. coli*	AMP, CIP, NAL				$bla_{\text{TEM-1}}$	
65.	60R	*E. coli*	AMP, CIP, NAL				$bla_{\text{TEM-1}}$	
66.	61R-R	*E. coli*	AMC, AMP, CHL, NAL, TET	+			$bla_{\text{TEM-176}}$	*qnrS1*
67.	62R-R	*E. coli*	AMP, CIP, NAL				$bla_{\text{TEM-198/214}}$	
68.	63R-R	*E. coli*	AMP, CFP, CRO, TET		+		$bla_{\text{CTX-M-15-like}}$	*qnrS1*
69.	63R-W	*E. cancerogenus*	AMC, FOX					
70.	64R	*E. coli*	AMP, CIP, NAL				$bla_{\text{TEM-1}}$	
71.	66R-R	*E. coli*	AMP, CIP, NAL				$bla_{\text{TEM-1}}$	
72.	66R-W	*E. cancerogenus*	AMC, AMP, FOX		+	+		
73.	67R-R	*E. coli*	AMP, CIP, CHL, NAL, TET	+			$bla_{\text{TEM-176}}$	*qnrS*
74.	67R-W	*E. cloacae*	AMC, AMP, FOX					
75.	69R	*E. coli*	AMP, CIP, NAL				$bla_{\text{TEM-1}}$	
76.	70R	*E. coli*	AMP				$bla_{\text{TEM-1}}$	

List of abbreviations: AMC, amoxicillin-clavulanic acid; AMK, amikacin; AMP, ampicillin; AmpC, AmpC beta-lactamases; CFP, cefoperazone, CHL, chloramphenicol; CIP, ciprofloxacin; CRO, ceftriaxone; ESBL, extended-spectrum β-lactamases; FOX, cefoxitin; GEN, gentamicin; MDR, multidrug resistance; NAL, nalidixic acid; TET, tetracycline. + indicate presence of the phenotype.
